# ‘Consciousnessoids’: clues and insights from human cerebral organoids for the study of consciousness

**DOI:** 10.1093/nc/niab029

**Published:** 2021-10-27

**Authors:** Andrea Lavazza

**Affiliations:** Centro Universitario Internazionale, Via Garbasso, 42, Arezzo 52100, Italy; University of Pavia, Department of Brain and Behavioural Sciences, Piazza Botta, 11, Pavia 27100, Italy

**Keywords:** states of consciousness, disorders of consciousness, anesthesia, computational modeling, theories and models

## Abstract

Human cerebral organoids (HCOs) are an *in vitro* three-dimensional model of early neural development, aimed at modelling and understanding brain development and neurological disorders. In just a few years, there has been a rapid and considerable progress in the attempt to create a brain model capable of showcasing the structure and functions of the human brain. There are still strong limitations to address, including the absence of vascularization that makes it difficult to feed the central layers of organoids. Nevertheless, some important features of the nervous system have recently been observed: HCOs manifest electrical activity, are sensitive to light stimulation and are able to connect to a spinal cord by sending impulses that make a muscle contract. Recent data show that cortical organoid network development at 10 months resembles some preterm babies’ electroencephalography (EEG) patterns. In the light of the fast pace of research in this field, one might consider the hypothesis that HCOs might become a living laboratory for studying the emergence of consciousness and investigating its mechanisms and neural correlates. HCOs could be also a benchmark for different neuroscientific theories of consciousness. In this paper, I propose some potential lines of research and offer some clues and insights so as to use HCOs in trying to unveil some puzzles concerning our conscious states. Finally, I consider some relevant ethical issues regarding this specific experimentation on HCOs and conclude that some of them could require strict regulation in this field.

## Introduction

The search for neural correlates of consciousness (NCCs) is a difficult undertaking, so far pursued in many ways and in many directions. One of the greatest difficulties (together with that of having a clear and shared definition of what consciousness is) generally derives from the fact that consciousness can only be detected indirectly, primarily through systematically collected verbal reports in human beings. The NCCs can be inferred from the correlation or even better from the identification of direct causation between the verbal reports that accompany subjective states and neuronal activity (Goldman 2000; Koch *et al.* 2016).

In the perspective I wish to develop here, one of the main problems of the search for NCC is the current impossibility to use one of the typical experiments of science, that of reproducing the phenomenon at hand. We know that every day in the world almost 400 000 children are born, who have the potential, and the vast majority of them do, to develop the consciousness that we recognize in a healthy adult human being. However, we are not able to understand how from the union of the two egg cells that give rise to the new organism including the brain the phenomenon of consciousness appears in the interaction of individuals with their environment. We do not know precisely what the necessary and sufficient elements and/or conditions are, and the goal of all the scientific community working on human consciousness is precisely discovering those elements and conditions.

The scientific study of the emergence of the phenomenon of consciousness from foetal brain development and in the perinatal phase is very complex and is not necessarily fundamental for understanding the functioning of consciousness (or at least we do not know if it is the case). Recent studies have started recording neural activations of the foetus related to, for example, a sound stimulus. Moser *et al.* (2019) investigated whether information-based metrics (measures of entropy, compressibility and fractality) of neural activity [detected using foetal magnetoencephalography (MEG) in human foetuses and neonates] are a useful tool for the quantification of consciousness before and shortly after birth. However, we are still in a preliminary methodological stage. Many obstacles (technical but also ethical, as we will see) seem to be overcome to get a better understanding of brain development in the human being connected to consciousness starting from conception to the early stages after birth, although certainly great steps forward have been made.

Now, a recent development in biomedical research, ‘organoids’, aimed at obtaining models for the study of many diseases and potentially to have parts of the organism to be transplanted without the problems of immune rejection, seems to be able to pave the way for a new and complementary approach to study consciousness and its neural correlates. The so-called organoids are stem cell–derived three-dimensional (3D) culture systems capable of recreating the architecture and physiology of human (and not only humans) organs in very good detail (Kim *et al.* 2020). More precisely, organoids are cell cultures grown in the laboratory that can mimic the spatial morphology, structural features and physiological responses of the represented organ of origin, as well as some of its key cell types. The first published landmark study on intestinal organoids dates back to 2009 (Sato *et al.* 2009). The first study reporting that cerebral organoids were grown was published in 2013 (Lancaster *et al.* 2013; Lancaster and Knoblich 2014).

Human cerebral (or brain) organoids (HCOs) have so far been grown and studied in order to have brain surrogates that can replace two-dimensional cultures and provide models for the study of pathologies and for the understanding of cellular mechanisms. In the making of the studies, further clues have emerged on the morphogenesis of the human nervous system, but the idea of creating from scratch a perfectly functioning human brain is not on any researcher’s agenda. The possible awareness of HCOs has only begun to be talked about by neuroethicists within a perspective that includes the precautionary principle (Cheshire 2014; Farahany *et al.* 2018; Lavazza and Massimini 2018a).

The goal of this paper is to bring to the attention of the community of consciousness experts the state of the art of HCO studies as regards the features that may affect the NCC search and to provide some first clues and insights concerning some possible strands of research on consciousness starting from HCOs. This does not mean that cerebral organoids are conscious today or that they necessarily will be in the future. At the current state of the techniques available for the growth of HCOs, we cannot even say with certainty that from the development and functioning of cerebral organoids we might understand the origin of human consciousness, although HCOs are brains grown in the laboratory that we can manipulate in an unprecedented way.

The Human Brain Project, whose aim is to replicate the working of the brain on a computer, has long been surrounded by scepticism: the *in vivo* study of the brain is unlikely to be replaced with *in silico* simulations. In the same way, it can be expected that the use of organoids in the laboratory will not be able to completely replace non-invasive experiments on humans. However, cerebral organoids could also become a first direct test bed for neuroscientific theories on consciousness currently on the market, and yet, we cannot exclude that HCOs may turn out to be unique entities from the point of view of consciousness.

Finally, some ethical issues concerning the research of NCC in HCOs will be discussed. In particular, based on the precautionary principle, it would be very ethically disputable to manipulate human organoids that are suspected of being or becoming partially conscious, thanks to new and more effective biomedical technologies. It might therefore be advisable to rather work on the cerebral organoids of primates. However, also this line of research might be objectionable by people who are against the use of animals for experimentation (Beauchamp and DeGrazia 2019). Indeed, cerebral organoids of primates could become conscious and feeling pain or distress as well as HCOs.

The structure of the article is as follows. In the ‘Physiology of cerebral organoids’ section, I describe the rapid progress that has led researchers to obtain small-sized cerebral organoids with high structural and functional complexity, the main features of current HCOs that are relevant to the study of consciousness and the potential that HCOs may have in this field in the short term and the medium term. In the ‘Cerebral organoids and consciousness’ section, I describe potential ways of measuring the spontaneous activity of cerebral organoids; I also provide some examples of how organoid research can aid NCC research, potentially shedding light on the origin of consciousness in the human brain, and I introduce the possibility of creating ‘consciousnessoids’ by assembling separately grown brain regions. In the ‘Ethics of consciousnessoids’ section, I consider potential ethical issues that might arise from using HCOs to study the mechanisms of consciousness. Some of these issues seem to be particularly challenging and could constitute a major limitation to research. In the ‘Conclusion’ section, I recapitulate the main points touched upon in the article with a look to the future.

## Physiology of cerebral organoids

Starting from embryonic stem (ES) cells or induced pluripotent stem (iPS) cells, it is possible to generate 3D *in vitro* cultures that mimic the developmental process and organization of the developing human brain (Arlotta and Paşca 2019; Setia and Muotri 2019). These HCOs have immediately provided a unique, physiologically relevant *in vitro* model system for the study of human neurological development and diseases. In general, organoids contain many different types of cells specific to the organ in which they are induced to develop, in order to reproduce the functionalities of the organ itself. This is achieved by using appropriate signalling factors that mimic the signalling environment typical of organ development in the human body. For this reason, organoids display a complex architecture similar to that observed *in vivo*: for example, HCOs have an appropriate cellular stratification.

Organoids have quickly found a wide variety of applications, from basic research to translational and industrial uses. First, organoids are providing important information on the development of the tissue that they model. Secondly, they also represent relevant models for studying cell biology, which includes tissue regeneration mechanisms and interactions with bacteria, viruses and cells from other tissues. From an experimental point of view, organoids add up the advantages of high complexity of cell cultures with the absence of confounding variables typical of animal models and the ease of *in vitro* handling and lower costs in terms of resources and time.

For this reason, in many cases (although not all), they can already complement or replace *in vitro* experiments that today use primary cells or immortalized cells and animal experimentations. The fact that organoids are genetically stable, i.e. they maintain the genotype and phenotype of the tissue of origin, allows them to be used as reliable models for the study of diseases and their mechanisms and progression. Organotypes are also being used to predict a patient’s specific response to a certain drug treatment. At least for some organs, it is possible to envision reaching, one day, the goal of what is considered the holy grail of biomedical research, namely the production of organs grown in the laboratory that can be transplanted into the body of a patient with full biological and immune compatibility, without needing a living donor.

An HCO is grown in the laboratory starting from an embryoid (tissue that has some embryonic features) obtained from ES or iPS cells. In the first experiment, ‘a combination of embedding in Matrigel as a polymerized gel, optimized media transitions and agitation of tissues led to the formation of cerebral organoids with various brain regions identities and discrete progenitor zones’ (Lancaster *et al.* 2013).

In general, the nervous system grows from the ectoderm layer of an embryoid. Ectodermal cells are placed into matrigel droplets (which provide nutrients) and floated in a nutrient broth in a rotating bioreactor. After 10 days, the organoid develops neurons. After 30 days, it displays regions similar to parts of developing brains in foetuses. Lacking vascularization and consequently blood and nutrient supply, brain organoids can reach about 4–5 mm across and remain vital for a year or even more, but they tend to have necrosis in the core due to hypoxia. For different scientific purposes, scientists grow 3D cell cultures systems of different complexity. They range from neurospheres (small clusters grown in suspension) to neural aggregates (based on pluripotent stem cells by first forming an embryoid body), up to cortical spheroids (containing only cortical neurons and astrocytes) and cerebral organoids or whole-brain organoids that are models derived from pluripotent stem cells capable of producing organized structures resembling those of the human brain.

By adding signalling and patterning factors [such as transforming growth factor-$\beta$ (TGF-$\beta$), bone morphogenetic protein (BMP), Wingless and Int-1 (WNT), fibroblast growth factor (FGF), sonic hedgehog (SHH), retinoic acid (RA) and extracellular matrix (ECM)], one obtains models of specific regions (e.g. the forebrain), while without patterning factors one obtains a complex structure representing multiple brain regions. In this sense, organoid protocols can be now classified as directed or guided and undirected or intrinsic. ‘Brain organoids recapitulate many features of the foetal human brain, including cytoarchitecture, cell diversity and maturation’ (Chiaradia and Lancaster 2020). Even though HCOs seem to recapitulate brain development up to 24 weeks, they lack endothelial cells and the co-presence of all the glial cell types.

This technique developed by Lancaster and colleagues and advanced also by other research groups (Velasco *et al.* 2019; Giandomenico *et al.* 2021) has already been used for the study (and treatment) of many diseases, starting from microcephaly and Zika virus to Angelman’s disease and Hungtington’s disease (Schwartz *et al.* 2015; Li *et al.* 2016; Qian *et al.* 2016, 2017; Yin *et al.* 2016; Pacitti *et al.* 2019; Sun *et al.* 2019; Grenier *et al.* 2020).

These important clinical applications go hand in hand with the possibility of studying the brain in all its development stages in unprecedented ways. Brain organoids that have been grown for many months have reached important levels of differentiation and cellular activity. The small spheres that initially appeared to be only a 3D transposition of the cultures of nerve cells on the Petri dish have begun to show important functionalities, and new advances may soon improve elements or features of organoids that still cannot be grown in the laboratory.

For example, Xiang *et al.* (2019) engineered human embryonic stem cells (hESC)s to ectopically express human ETS variant 2 (*ETV2*). *ETV2*-expressing cells in HCOs contributed to forming a complex vascular-like network. Importantly, the presence of vasculature-like structures resulted in enhanced functional maturation of organoids (Xiang *et al.* 2019). Pellegrini *et al.* (2020) managed to establish human ChP organoids with a selective barrier and cerebrospinal fluid–like secretion in self-contained compartments. This is another relevant advance in growing HCOs that are more similar to *in vivo* brains.

Many features of HCOs are central in many neuroscience subfields, but we are here interested in what is mostly related to consciousness study. As summed up by Chiaradia and Lancaster (2020), ‘the hallmark of neuronal maturation is the acquisition of spontaneous firing activities and the emergence of dendritic spines and synaptic contacts, enabling the transmissions of nerve impulses along the network. Both inhibitory and excitatory synapses have been observed in brain organoids, together with functionally relevant presynaptic vesicles’.

Just to name a few, Birey *et al.* (2017) produced ‘three-dimensional spheroids from human pluripotent stem cells that resemble either the dorsal or ventral forebrain and contain cortical glutamatergic or GABAergic neurons’, thus showcasing the saltatory migration of interneurons in the foetal forebrain. They also showed that after migration, interneurons functionally integrate with glutamatergic neurons to form a microphysiological system. ‘Spheroids cells were remarkably similar with those from corresponding regions of humans’ fetal brain’, with ‘both excitatory and inhibitory neuronal activity’ (Camp and Treutlein 2017; cf. Pacitti *et al.* 2019).

It is said that without inputs and outputs, the HCO’s neural networks cannot reach maturity, but the issue is open because ‘transcriptional analysis and comparison to the developing human brain have revealed that hCSs after 2.5 months resembled the mid-fetal prenatal brain (19–24 post-conception weeks). Cortical neurons were accompanied by a network of nonreactive astrocytes and were synaptically connected’ (Paşca *et al.* 2015; Paşca 2018). Today, laboratory-made cerebral organoids already ‘acquire structural traits of mature neurons, including dendritic spine-like structures’, and researchers have recorded excitatory spikes in organoids grown for 8 months, where monosynaptic connections were detected with high-density silicon microelectrodes (Quadrato *et al.* 2017). These findings ‘suggest that brain organoids establish neuronal networks that can support self-organized patterns of activity’ (Quadrato *et al.* 2017).

Also, HCOs show the differentiation of photoreceptor-like cells endowed with proteins for light responsiveness. These photosensitive cells ‘can respond to non-invasive, light-based sensory stimulation’ (Quadrato *et al.* 2017). Very recently, optic vesicle-containing brain organoids (OVB) have been grown (Gabriel *et al.* 2021). These HCOs are engineered to define primordial eye fields and progressively develop bilaterally symmetric optic vesicles and neural and non-neural cell types. Importantly, they are light-sensitive, and ‘various light intensities could trigger photosensitive activity of OVB-organoids (...). Thus, brain organoids have the intrinsic ability to self-organize forebrain-associated primitive sensory structures in a topographically restricted manner’ (Gabriel *et al.* 2021). These steps forward indicate that it is possible to transmit afferent stimulations to cerebral organoids, and this has important implications, since so far one of the main limitations in the development of HCOs has been precisely the fact that they do not have any sensory communication with their environment. A further step forward has been made with new methods of cultivation of cerebral organoids (air–liquid interface) that have allowed to generate diverse nerve tracts with functional outputs (Giandomenico *et al.* 2019). In this way, ‘these cultures exhibit active neuronal networks, and subcortical projecting tracts can innervate mouse spinal cord explants and evoke contractions of adjacent muscle in a manner dependent on intact organoid-derived innervating tracts’ (Giandomenico *et al.* 2019). In other words, cerebral organoids have proved capable of inducing movement, although not yet of a purpose-oriented kind.

A recent study showed for the first time that cortical organoids generated from iPS cells can spontaneously develop periodic and regular oscillatory network electrical activity that resembles the electroencephalography (EEG) patterns of preterm babies (Trujillo *et al.* 2019). This means that, even in the absence of external or subcortical inputs, 10-month-old HCOs can develop according to a specific genetic program, like all human beings, and manifest a complex brain activity recorded with multi-microelectrode array (MEA). ‘The spontaneous network formation displayed periodic and regular oscillatory events that were dependent on glutamatergic and GABAergic signaling’ (Trujillo *et al.* 2019). The firing rate, up to 2 or 3 per second, and the kind of waves—gamma, alpha and delta—are all a hallmark of a vital human brain. Indeed, a machine-learned model based on a preterm newborn’s EEG (ranging from 24 to 38 weeks) features was able to predict the organoid culture’s age based on the electrical activity of the organoid itself. In other words, the software found no significant differences in EEG between patterns of preterm babies and patterns of HCOs. These results, although very relevant, do not mean the recorded patterns of activity give rise to the same subjective states as that can be believed to have originated in preterm babies, such as pain sensations that foetuses after 24 weeks can likely experience.

In another study (Sakaguchi *et al.* 2019), researchers have managed to visualize in cortical spheroids synchronized and non-synchronized activities in networks and connections between individual neurons. They managed to detect dynamic changes in the calcium ion activity and find comprehensive activities among cells capable of organizing themselves into clusters and form networks with other nearby clusters. The manifestation of a synchronized neural activity can be the basis for various relevant brain functions, including memory. Another important element brought to light by research is that neurons grown *in vitro* fire spontaneously, which is one of the ways neurons grow and create new connections.

It is known that neural activity in cortico-striatal circuits of the forebrain and projections from it are central in coordinating motivated behaviours and movement and that the ventral striatum has been considered as a relevant region for consciousness (Slagter *et al.* 2017). To enable the study of the human cortico-striatal pathway, Miura *et al.* (2020) developed a method to convert human pluripotent stem cells into region-specific brain organoids that resemble the developing human striatum and include electrically active medium spiny neurons. The group led by S. P. Paşca succeeded in assembling striatum organoids with cerebral cortical ones in 3D cultures to form cortico-striatal assembloids. ‘Using viral tracing and functional assays in intact or sliced assembloids, we show that cortical neurons send axonal projections into striatal organoids and form synaptic connections. Medium spiny neurons mature electrophysiologically following assembly and display calcium activity after optogenetic stimulation of cortical neurons’ (Miura *et al.* 2020).

Indeed, a specific technology (Marton and Paşca 2020) allows to combine organoids resembling distinct areas into assembloids and can be used to model aspects of interactions that occur between regions in the human brain. Organoids can also be supplemented with non-central nervous system-derived cell types, including microglia and endothelial cells, to study the interplay of nervous system cells with immune cells and blood vessels (cf. also Xiang *et al.* 2019).

Recently, Paşca and his group succeeded in deriving organoids resembling the cerebral cortex or the hindbrain/spinal cord and assemble them with human skeletal muscle spheroids to generate 3D three-component cortico-motor assembloids (Andersen *et al.* 2020). First, the components of a cortico-motor circuit were grown separately and then functionally integrated, thanks to the connections that region-specific spheroids form when they are assembled. In this way, neurons of the cerebral cortex were connected through descending pathways to the hindbrain and the spinal cord to activate muscles and generate movement via motor neurons.

Importantly, Fair *et al.* (2020) investigated the developmental trajectory of electrophysiological properties (EPs) in whole-brain HCOs and correlated these properties with developmentally linked morphological and cellular features. The authors used a 64-channel MEA platform to detect and record spontaneous extracellular field potential change activity. They noted a gradual evolution of EP features in HCOs within 5 months in cultures. ‘Maturation of electrical features correlated with dynamic changes in the development of cell types within COs, such as the emergence of astrocytes and diverse neuronal populations. Last, as COs transition into increased cellular and morphological complexity, we observed activation of the neurotrophin (NTR)/TRK receptor signaling pathway’ (Fair *et al.* 2020).

The study seems to show that HCOs have ‘a gradual evolution of EP properties over development that resembles hallmark features of the developing neonatal brain’ (Fair *et al.* 2020). Furthermore, an increase in the cellular diversity of HCOs and a correlation with their EP trajectories have been observed, where the presence of inhibitory neurons in late-stage HCOs can indicate the maturation (also at the protein and transcriptome levels) of local cortical circuitry within neural networks. Another relevant element is the presence of GABAergic neurons and a diversity of apical and basal radial glial subtypes.

## Cerebral organoids and consciousness

Some of the most promising advances in organoid technology have been published in the last 30 months, showcasing a notable acceleration, thanks to the possibility of creating living assembloids. Also, several research institutions are working and investing in brain organoids. Research on HCCs can therefore be said to be on the fast rise, and we can easily guess that we will have increasingly ‘perfected’ organoids in the near future. Bioengineering (Garreta *et al.* 2021) promises to overcome some well-known shortcomings organoids still exhibit, such as lack of specificity with regard to cell-type compositions, uncontrolled size, shape heterogeneity, absence of proper vascular and immune components and organ-specific morphological features, and absence of some kinds of genetic expression.

Obviously, we do not know if the vascularization problem, which is the key to having larger HCOs through oxygen and nutrients supplementation, will be solved and possibly when, while the possibility of inducing well-organized regional identities seems closer (Mansour *et al.* 2018; Çakir *et al.* 2019; Garreta *et al.* 2020). Useful tools could be 3D printers, microfluidic devices, bioreactors and robotic devices. Even optogenetics will be a technique that will allow the neuronal activity of brain organoids to be finely guided as in humans for safety reasons it is not yet possible (Shiri *et al.* 2019; Yoon *et al.* 2019). New 3D interfaces will allow one to study HCOs with the highest precision (Park *et al.* 2021).

However, it should be emphasized that so far research has focused on qualitative aspects, so to speak, of brain organoids, that is, we have tried to obtain specific regions or specific functions so to try to find the origin of disorders or diseases or to try to understand the specific stages or moments of neurogenesis. In order to study the emergence of consciousness, it will probably be necessary to focus on whole-brain organoids and also on quantitative aspects (we are talking about some hundreds million-neuron brains grown in the laboratory versus an 86 billion-neuron adult brain). The overall size and total number of neurons could certainly be decisive at least below a certain threshold. On the other hand, if we believe that consciousness comes in degrees, we might think that the qualitative and quantitative development of the brain allows us to move from mere sentience (awareness consciousness) to the ability to experience affective states of specific valence (phenomenal consciousness) up to self-consciousness.

But this hypothesis could be falsified precisely by empirical studies on HCOs, as thinking that the ontogenesis of the nervous system simply summarizes phylogeny is probably a mistake that comes from outdated theories. In any case, identifying a threshold of neural development in terms of size, cellular differentiation, connectivity and activity of specific circuits could be a pivotal turning point itself and for the ethical and pragmatic implications.

In the light of current knowledge on HCOs, I would like to show here only some potential strands of research, although some of them could soon turn out to be dead ends (cf. Bayne *et al.* 2019). The aim is to elicit more insights and to foster a theoretical debate and an expansion of experiments with HCOs so as to include the basic study of consciousness mechanisms. This expansion of the experiments should be accompanied by a high sensitivity to ethical issues.

In subsection ‘Assessing cerebral organoid activity’, I present a method to infer the potential presence of consciousness in laboratory-grown cerebral organoids. This may allow the advancement of research on consciousness even on the basis of systematically negative results. In subsection Organoid properties relevant to understand consciousness, I introduce some aspects related to the organoid properties that could be potentially relevant to understanding consciousness. In subsection Cerebral organoids as potential ‘consciousnessoids’, I propose some ideas for specific experiments that could provide new insights into the emergence and functioning of consciousness.

### Assessing cerebral organoid activity

The first step to advance in the study of consciousness through cerebral organoids is to record their spontaneous and induced activity. As we have seen, adequate EEG equipment is already available in this sense. The analysis of the tracing thus obtained can provide interesting, but not conclusive, elements for the assessment of possible forms of sentience/consciousness (cf. Trujillo *et al.* 2019). A more complex and promising type of analysis is the Perturbational Complexity Index (PCI), a metric that is inspired by the main postulate of Integrated Information Theory (IIT), that is, that consciousness relies on the joint presence of integration and differentiation in neural circuits (cf. Tononi and Sporns 2003; Tononi 2008; Tononi and Koch 2015; Tononi *et al.* 2016).

Calculating PCI involves locally perturbing the cerebral cortex through transcranial magnetic stimulation (TMS) and measuring the complexity of the electrical response of the rest of the brain with electroencephalography (Massimini *et al.* 2009; Casali *et al.* 2013; Gosseries *et al.* 2014; Sarasso *et al.* 2014). The rationale is that PCI should be low if interactions among neural elements are reduced due to the loss of integration, because the response engaged by TMS is spatially restricted; PCI is also low if many interacting areas react to the perturbation in a stereotypical way due to loss of differentiation, because in this case the resulting response is large but simple. PCI should reach high values only if the initial perturbation is transmitted to a large set of neural elements that react in a differentiated way (cf. Lavazza and Massimini 2018a).

Being based on general theoretical principles, PCI is totally independent of sensory processing, executive functions or motor behaviours and can be graded. Since brain-injured, unresponsive patients are fully inaccessible and do not provide any reliable evidence about their state of consciousness to be used on them, PCI had to be first validated and calibrated on a large benchmark population of subjects who could confirm the presence or absence of conscious experience through reports. Despite some individual variability within this large sample, PCI was lower in all unresponsive subjects who did not report any conscious experience on awakening from non-rapid eye movement (NREM) sleep or midazolam, xenon and propofol anaesthesia and was invariably higher in conditions in which consciousness was present (Casarotto *et al.* 2016).

An advanced version of PCI, possibly using finer stimulation and recording techniques (a combination of optogenetic stimulation and calcium imaging), may be developed in the future for cerebral organoids—a version adapted for *in vitro* cortical slices has been tested already (D’Andola *et al.* 2018). Clearly, the problem would still be how to validate this new index and identify a valid operational cut-off above which we could establish that the cerebral organoid has some capacity for consciousness. As for PCI, the cut-off determination process would, however, need to start from some known points of reference, for example, the values exhibited in the brain of an adult human being across different states (wakefulness, sleep, dreaming, anaesthesia and brain injury) and then gradually move to more challenging cases, such as newborns, primates, rodents and finally organoids. To the extent that the proposed measurement (a potential novel index of network complexity) is good enough to be generalized across species and types of brain circuits, it could at least allow for a coarse comparison on a common scale.

Although initially inspired by the Integrated Information Theory of consciousness (which is not unanimously considered a sound theory, e.g., Merker *et al.* 2021), the PCI index is recognized as a potential general indirect measure of the presence of consciousness. If properly developed and validated, PCI would make it possible not only to ascertain the presence of consciousness in HCOs but also to advance research by working on negative results. In fact, on studying the different development degrees of brain organoids, one would be able to either ascertain or exclude that consciousness emerged at a given stage and/or under given conditions. As the assembloids progress and the sensory and effector channels mature, PCI would allow us to assess the progressive degrees of integration of the overall activity of brain organoids, enabling us to understand the relative contribution of the various regions involved at different development stages and at different degrees of complexity of the HCOs analysed.

Very recently, Ankeny and Wolvetang (2021) proposed that

the most fruitful strategy to benchmark human brain organoids will be to apply technologies and measures currently used to assess levels of consciousness in comatose human patients and non-human animals. Since consciousness is strongly correlated with irregular low-amplitude electroencephalographic (EEG) activity in the 20–70 Hz range as well as gamma wave synchrony between different brain regions, measuring these parameters in organoid and combinoids with multi-electrode arrays would be an important first step.

In addition, they advocated ‘assessing whether the bispectral index (BIS), which combines different features of the EEG to gauge anesthetic depth in anesthetized patients (Myles *et al.* 2004), can be performed in human brain organoids and would be useful, complemented with other types of analyses that mimic protocols increasingly used to measure stimulus-evoked activity in vegetative and minimally conscious patients’. Also, MEG could be a good method for making a comparison between magnetic fields generated by the electrical activity in cerebral organoids and those in the brains of foetuses or comatose patients (Gross 2019).

### Organoid properties relevant to understand consciousness

At this stage, one can wonder what type of study is required when it comes to organoids, and why it is relevant to our understanding of consciousness. A potential path in this sense is shown by Silva *et al.* (2020). In their opinion, ‘physical constraints imposed on the brain can guide the analyses, an interpretation of experimental data and the construction of mathematical models that attempt to make sense of how the brain works and how cognitive functions emerge. Development of these mathematical models for human-derived brain organoids offers an opportunity for testing new hypotheses about the human brain’.

If the computational space is finite, it is limited by the physical constraints imposed on the brain. All theoretical or computational models should take into account a fundamental structure–function constraint, which is the result of interaction between anatomical structure and signalling dynamics: ‘It is a constraint produced by the way the brain is wired, and how its constituent parts necessarily interact’ (Silva *et al.* 2020).

In this vein

Human-derived brain organoids reflect a personalized model of the brain unique to each individual. Because of this, they could provide an opportunity to bridge biological experiments and computational models with behavioral, cognitive, and clinical studies in humans that no other experimental model is able to achieve. Organoids derived from neurotypical individuals or patients can be experimentally studied in parallel with cognitive experiments or clinical trials being carried out in the same individuals. They can potentially put molecular, cellular and physiological scale experiments distinct to each individual in context with cognitive and clinical studies that the subjects or patients are participating in. This would be invaluable for the investigation of physiologically complex genetically based neurodevelopmental and neurological disorders such as autism spectrum disorder and epilepsy (Silva *et al.* 2020).

Accordingly, some relevant neuroscientific issues might be addressed. For instance, consider the connectivity structure of the brain, which is strongly linked to genetic make-up. Is it formed in a stochastic way or is it wired purposefully? As for the neural dynamics of consciousness, a more pertinent observation is that in artificial simulations of a biological network, the wrong kind of geometry and connectivity can easily destroy the dynamic activity of the network (Buibas and Silva 2011).

This perspective of experimental testing on HCOs also encompasses computational theories of consciousness, such as Higher-Order Thought theories, or the Higher-Order Syntactic Thoughts approach, which aims to ‘identify the computations that are linked to consciousness, and to analyze the neural bases of those computations’ (Rolls 2020). Another related theory in this sense is the Global Neuronal Workspace Model, when used as a computational theory of conscious processing (Dehaene *et al.* 2014).

On the other hand, some cellular mechanisms of conscious processing have recently been highlighted. These are the first candidates for a study that moves from animal models to human brain models consisting of HCOs. In particular, the research carried out by Aru and colleagues has noted the importance of the biophysical properties of pyramidal cells. The latter are believed to constitute ‘gates that control the evolution of global activation patterns’, within the thalamocortical system. Aru proposed a Dendritic Information Theory, which is a neurobiological theory of consciousness, whose hallmark ‘is the flexible integration of bottom-up and top-down data streams at the cellular level’ (Aru *et al*. 2020).

The study of the effects of anaesthesia has led to the inference that cortical pyramidal neurons may play a key role in the mechanisms of consciousness, although the latter is thought to be a property of activity patterns distributed over large brain networks. L5p cells have distinct functional compartments that facilitate the segregation and recombination of multiple input streams. The middle compartment, called ‘coupling compartment’, mediates interaction between the apical and basal compartments. In unconscious states, the apical compartment seems to be unable to influence basal compartments and this decoupling effect might be what triggers the loss of consciousness (specifically in anaesthesia; Suzuki and Larkum 2020).

The dual-stream information flow gated by L5p neurons is compatible with computational theories such as predictive coding based on the principle of free energy minimization (Friston *et al.* 2017). However, these theories still need to be tested and, as mentioned, brain organoids lend themselves to providing the right physical constraints to do so. In general, some of the claims of the Dendritic Integration Theory and the questions that its proponents raise for future studies (Aru *et al*. 2020) could find experimental answers through the use of specific human brain models consisting of organoids. In particular, the non-invasive manipulation and measurement of decoupling is extremely complex in animal models and currently impossible in the brains of adult human volunteers. In this sense, organoid research on consciousness could represent a major breakthrough.

### Cerebral organoids as potential ‘consciousnessoids’

When it comes to directly probing or using HCOs as a platform for consciousness research, one of the first steps may be to exclude from the analysis brain areas and patterns of neural activity that in the typically developing brain have turned out to be probably unrelated to the emergence of consciousness. Secondly, the issue of dimensions and the lack of sensory inputs should be considered. Regarding the first aspect, the Global Neuronal Workspace Theory (Dehaene and Naccache 2001; Dehaene *et al.* 2006, 2011; Dehaene 2014) posits that one becomes conscious of something only if unconscious brain activity in sensory areas spreads to a larger network of neurons throughout the brain via long-distance connectivity with specific cortico-cortical architecture. Regarding the second aspect, approaches such as the Temporo-Spatial Theory of consciousness proposed by Northoff presuppose an integration of sensory stimuli as a basic element of human consciousness (Northoff and Huang 2017; cf. Zilio 2020).

If it makes no sense to directly test theories on consciousness, which have very specific assumptions concerning an adult human brain in interaction with its environment, one potential way is precisely the engineering of neural systems that reproduce the features of structure and connectivity considered as necessary for the emergence of consciousness (Varrault *et al.* 2019). For example, regarding global workspace theory, creating assembloids (see ‘Physiology of cerebral organoids’ section) capable of mimicking the network deemed to constitute the minimum necessary NCCs would allow a new and more precise kind of experimentation. But one should not forget that these potential ‘consciousnessoids’ would have the limit of not being able to give a behavioural (nor obviously verbal) output based on which to verify the correlation between NCCs and experienced subjective states. And yet, as will be said further on, an assembloid that also has rudimentary effectors does not seem impossible to grow.

Another line of research is that which uses firing patterns and connectivity benchmarks believed to be reliably correlates of conscious or unconscious states to test different neural signatures (Dehaene and Changeux 2011). In this case, if strong similarities were discovered, inferences of scientific interest could be made, although there would still be no direct evidence on the presence of consciousness in the brain organoids examined. Some recent studies that can provide insights for studies on the activity in organoids concerns specific patterns of neural activation detected in altered states of consciousness and in different phases of sleep.

The findings in Pigorini *et al.* (2015) ‘suggest that the intrinsic tendency of cortical neurons to fall into a down-state after a transient activation (i.e., bistability) prevents the emergence of stable patterns of causal interactions among cortical areas during NREM [sleep]. Besides sleep, the same basic neurophysiological dynamics may play a role in pathological conditions in which thalamo-cortical information integration and consciousness are impaired in spite of preserved neuronal activity’. Another well-known study investigated potential confounding factors concerning physiological variables which change when subjects pass from wakefulness states to sleep. Nieminen *et al.* (2016) found evidence which seems to show that variations in the level of consciousness within the same physiological state are associated with changes in the underlying bistability in cortical circuits.

Subsequently, Siclari *et al.* (2018), stated that ‘in both NREM and REM sleep, reports of dream experience were associated with local decreases in low-frequency activity in posterior cortical regions. High-frequency activity in these regions correlated with specific dream contents. Monitoring this posterior “hot zone” in real time predicted whether an individual reported dreaming or the absence of dream experiences during NREM sleep, suggesting that it may constitute a core correlate of conscious experiences in sleep’.

In this sense, the first step could be to compare the functional activity that can be recorded in cerebral organoids (for example both with MAE techniques and with calcium-imaging-based methods to have the maximum possible resolution both in space and time) with benchmarks of conscious activity of human beings in different stages of the development of the nervous system both in specific states of consciousness (based on the age of development) and in states of altered consciousness (anaesthesia, coma, vegetative states).

The inferences that could be drawn from this comparison should obviously extend from the electrical activity to the architecture of the circuit that generates it up to the analysis of genes that are specifically expressed in the neural activations considered (Tanaka *et al.* 2020). Concerning these features, an automated multiscale comparative analysis dubbed SCOUT was recently proposed. This ‘integrated technology platform can rapidly clear, label, and image intact organoids. Algorithmic- and convolutional neural network-based image analysis extract hundreds of features characterizing molecular, cellular, spatial, cytoarchitectural, and organoid-wide properties from fluorescence microscopy dataset’ (Albanese *et al.* 2020).

Any overlap between activity recorded in the cerebral organoids and the chosen benchmarks would in any case not give confirmation of glimpses of consciousness in the absence of interaction with the environment but would still give important preliminary information. Subsequently, one could begin to think of first attempts at experimentation on organoids aimed directly at testing aspects at least potentially related to consciousness. Given that the possibility of growing HCOs that are sensitive to light stimulation has already been ascertained, one could think of evaluating the type of neural activity that is elicited by different wavelengths, corresponding to different colours and comparing this activity with the preference for different colours manifested by a control group of infants and with the mean neuronal activity corresponding to each colour in the control group.

In the strand of studies on assembloids, one could try to combine a cortico-motor assembloid with another component that includes pain sensitive sensors, also of animal origin, and evaluate whether the administration of generally painful stimuli, such as strong heat, can trigger a specific activity at the motor level (however you want to interpret it). Nerve terminations sensitive to stimuli and rudimentary effectors that give feedback in terms of peculiar neural activation could be the first tests carried out directly on cerebral organoids.

On a different level, in the debate on the brain-likeness of HCOs, it has been interestingly proposed to consider memory as something that is specific to the nervous system, for which there is biological evidence, the mechanisms of which are rationally explainable and measurable (Lunshof 2021). The questions that we could then ask ourselves in the laboratory are as follows: ‘does HCOs possess the neurobiological features necessary for extant memories?’ and ‘can we endow an engineered cerebral organoid with memories?’. Memory can be a proxy of the presence of consciousness, but it seems to be linked to the ability to receive sensory inputs. In any case, even the manifestation of a behaviour guided by information retrieved from the memory does not necessarily show the presence of consciousness, as this behaviour can be ‘automatic’ or otherwise controlled by subpersonal processes.

A recent experiment can be considered in this sense. Vetere *et al.* (2019) managed to form a memory in the absence of experience, thanks to optogenetics. Since ‘memory is coded by patterns of neural activity in distinct circuits’, it is feasible to reverse engineer a memory by artificially creating specific patterns of activity directly affecting the neuronal activation. In the experiment conducted on mice, ‘in olfactory conditioning, an odor conditioned stimulus (CS) is paired with an unconditioned stimulus (US, for example, a footshock), and the resulting CS–US association guides future behavior’.

We replaced the odor CS with optogenetic stimulation of a specific olfactory glomerulus and the US with optogenetic stimulation of distinct inputs into the ventral tegmental area that mediate either aversion or reward. In doing so, we created a fully artificial memory in mice. In a similar way to a natural memory, this artificial memory depended on CS–US contingency during training, and the conditioned response was specific to the conditioned stimulus and reflected the unconditioned stimulus valence. Moreover, both real and implanted memories engaged overlapping brain circuits and depended on basolateral amygdala activity for expression Vetere *et al.* (2019).

From this experiment, we can deduce that the brain does not need an external experience to create a memory, although obviously we are talking about a very simple association between a sensory datum and a pleasant or painful stimulation. Somehow, we now know that brains in a vat are not just a logical possibility, being the reference to the thought experiment made famous by philosopher Putnam (1981), about the eventuality that our entire life is a simulation experienced by a brain immersed in a bath of nutrients and connected by cables to a computer.

An intervention of the type described by Vetere could also be produced on a cerebral organoid of adequate size, in order to verify whether it is possible to reproduce the same or similar neuronal pathways and the same or similar activations, thanks to the use of optogenetics, which turns out to be the ideal tool for experiments of this kind also in HCOs. Provided it can be agreed that having the memory of an unpleasant experience has some effect on the mouse, its behavioural response seems to confirm the effect of this sensation. In the case of the cerebral organoid, this check could not be obtained directly, but if the circuit of a memory could be created in the absence of experience, the need for sensory input to the nervous system and probably also for a body would disappear. In fact, if the memory of the unpleasant experience is stored in the engram without the need for sensitivity to the external environment, then it can also be relived as unpleasant without a body and afferent nervous pathways.

The obvious objection is that the activation of the circuit that produces aversion to a certain environment in the mouse based on the unpleasant memory may not produce any conscious sensation in the cerebral organoid. A possibility is using the above-mentioned PCI to verify what degree of integration is recorded when the aversion is manifested. However, whatever the result of the PCI test, it would not warrant that the memory created in the organoid will be a conscious one.

## Ethics of ‘consciousnessoids’

Is it possible that HCOs may show a form of sentience or a more advanced degree of consciousness? The overwhelming majority of scientists believe this is currently not the case. However, a number of neuroethicists have begun to consider this possibility, generally based on two types of consideration (Lavazza and Massimini 2018b; Hostiuc *et al.* 2019; Lavazza 2019, 2021; Sawai *et al.* 2019; Lavazza and Pizzetti 2020). The first is the factual observation that the development of scientific research on HCOs is progressing at a very fast pace, and many typical functions of the brain of a human being start being detected in brain surrogates grown in the laboratory. The second consideration concerns the precautionary principle, ‘which states that in situations of some types of uncertainty, a decision-maker should refrain from actions or policies that run the risk of causing harm to the public or to the environment, even if the harmfulness of these actions or policies has not been scientifically established beyond reasonable doubt’ (Żuradzki 2021).

In this vein, Birch and Browning (2021) claimed that ‘if an organoid contains structures or mechanisms that any serious and credible theory of the human NCCs posits to be sufficient for conscious experience, we should take proportionate measures to regulate research on that organoid. In practice, this sets the evidential bar for taking precautions at an intentionally low level with the specific qualities typical of the human being in the growth phase’. Greely (2021) chose an intermediate position and stated that ‘when we avoid unethical research by making living models of human brains, we may make our models so good that they themselves deserve some of the kinds of ethical and legal respect that have hindered brain research in human beings. If it looks like a human brain and acts like a human brain, at what point do we have to treat it like a human brain—or a human being?’.

On the contrary, Koplin and Savulescu (2019) have proposed to make the use of HCOs proportionate to some critically important purposes or sufficiently great expected benefits of the research. This view implies the lawfulness of using both ‘conscious or potentially conscious brain organoids (equivalent to 20 weeks’ in vivo brain development or more)’ and ‘brain organoids with the potential to develop advanced cognitive capacities (e.g., mature brain organoids capable of interacting with the outside environment)’. This framework to regulate the use of HCOs capable of developing higher consciousness and cognitive abilities is based on a consequentialist perspective that seems to admit a limited exploitation of HCOs in exchange for great expected benefits related to biomedical research. Hyun *et al.* (2020) take a very similar stance and do not consider cerebral organoids currently endowed with consciousness and that they should not therefore be attributed a moral status of some kind.

The moral consequentialist view taken by those who believe that research and destructive experiments on brain organoids are legitimate is based on their use in the biomedical field. If research on HCOs can save lives, it is said, it is certainly legitimate to exploit them for this purpose, whether or not they have a minimum degree of sentience, in other words, a sensitivity to pleasure and pain. In this article, I have instead dealt with a specific research that could be conducted with HCOs and that up to now has not yet been undertaken, namely that on NCCs.

In such a case, we would be faced with an unprecedented situation, as pointed out by Greely (2021). If we use HCOs to study consciousness and manage to make them grow so that they begin to show some form of consciousness, we should address a particularly difficult moral dilemma. As Lavazza (2020) pointed out: ‘Even considering HCOs as entities with a unique ontological status that needs to be clarified, they certainly share two convergent criteria for the attribution of moral status: the fact of potentially having a rudimentary form of consciousness and the fact of being part of the human species’.

And specifically

the use of cerebral organoids—if developed to very advanced stages precisely to study the emergence of human consciousness and its mechanisms— would end up violating Kant’s humanity formula in the extended formulation proposed here, which requires not using certain entities as means but only as ends. In this sense, even the mere culture of HCOs in the laboratory to do research on human consciousness would amount to a similar violation. In fact, even the recognition of a minimal form of moral status combined with Kant’s proviso induces to spare such an entity forms of suffering that it could experience as a sentient being (Lavazza 2020).

## Conclusion

In this paper, possibly for the first time, an attempt has been made to present some clues and insights that organoid research could provide in the search for the neural mechanisms and correlates of consciousness. HCOs are part of an expanding field, but until now they have been mainly used as a model for the study of neurological diseases and neurodevelopment. After describing some relevant features of the currently available organoids, I suggested that some recent findings and the development of new techniques could allow for extending the use of HCOs also to the study of consciousness.

In particular, first, I proposed the application of the PCI as a candidate for a suitable strategy to test the presence of proxies of conscious activity in HCOs. This route may allow research to progress even on the basis of systematically negative results. Second, I introduced some aspects related to the various organoid properties that are relevant to the understanding of consciousness, considering firstly the physical constraints and the structure–function approach and secondly some specific cellular mechanisms of conscious processing, namely pyramidal neurons. Then, I noted that the progress of assembloids and technologies aimed not only at growing organoids but also at engineering specific circuits and connections could lead to the creation of ‘consciousnessoids’. These organoids would be capable of mimicking the features of a neural system displaying the characteristics that various theories of consciousness take to be the minimum NCC for the emergence of conscious states. At that point, HCOs could become unprecedented living laboratories for the study of consciousness.

Finally, I have highlighted an important ethical issue. If HCOs manifested some form of consciousness or it could be indirectly assumed that they possess it, this would call for consideration of whether HCOs should be given a moral status and what limitations should be introduced to regulate research with HCOs. I proposed that using HCOs that were conscious would be a violation of the requirement not to use sentient beings of human origin as pure means.

In this vein, when the stage of minimally conscious HCOs was reached, a recommended step could be to study consciousness through brain organoids derived from non-human animals, looking for the developmental processes underlying the neurophysiological mechanisms that correlate with sentience in healthy adult animals (Kanton *et al.* 2019). In this case too, however, one might ask what ethical issues would be raised by those experiments, given non-human animals too deserve to bear the minimum suffering possible.

It therefore seems that research on consciousness thanks to cerebral organoids opens up extremely interesting potential new avenues but raises ethical issues that will not be easily solved.^1^

## Footnotes

1 I thank two anonymous reviewers very much for their helpful comments and Federico Zilio for insightful conversations and for his book (Zilio 2020), which was a relevant source for a paper of mine (Lavazza 2020).
